# Investigating the CYP2E1 Potential Role in the Mechanisms Behind INH/LPS-Induced Hepatotoxicity

**DOI:** 10.3389/fphar.2018.00198

**Published:** 2018-03-07

**Authors:** Hozeifa M. Hassan, Bashir A. Yousef, Hongli Guo, Liu Xiaoxin, Luyong Zhang, Zhenzhou Jiang

**Affiliations:** ^1^Jiangsu Key Laboratory of Drug Screening, China Pharmaceutical University, Nanjing, China; ^2^Department of Pharmacology, Faculty of Pharmacy, University of Gezira, Wad Medani, Sudan; ^3^Department of Pharmacology, Faculty of Pharmacy, University of Khartoum, Khartoum, Sudan; ^4^Jiangsu Center for Pharmacodynamics Research and Evaluation, China Pharmaceutical University, Nanjing, China; ^5^Center for Drug Screening and Pharmacodynamics Evaluation, School of Pharmacy, Guangdong Pharmaceutical University, Guangzhou, China; ^6^Key Laboratory of Drug Quality Control and Pharmacovigilance (China Pharmaceutical University), Ministry of Education, Nanjing, China

**Keywords:** isoniazid, lipopolysaccharide, hepatotoxicity, diallyl sulfide, dexamethasone, CYP2E1, apoptosis, steatosis

## Abstract

Tuberculosis (TB) is one of the oldest infectious diseases that affected humankind and remains one of the world’s deadliest communicable diseases that could be considered as global emergency, but the discovery and development of isoniazid (INH) in the 1950s paved the way to an effective single and/or combined first-line anti-TB therapy. However, administration of INH induces severe hepatic toxicity in some patients. Previously, we establish a rat model of INH hepatotoxicity utilizing the inflammatory stress theory, in which bacterial lipopolysaccharide (LPS) potentially enhanced INH toxicity. These enhancing activities ranged between augmenting the inflammatory stress, oxidative stress, alteration of bile acid homeostasis, and CYP2E1 over-expression. Although pre-treatment with dexamethasone (DEX) helped overcome both inflammatory and oxidative stress which ended-up in alleviation of LPS augmenting effects, but still minor toxicities were being detected, alongside with CYP2E1 over expression. This finding positively indicated the corner-stone role played by CYP2E1 in the pathogenesis of INH/LPS-induced liver damage. Therefore, we examined whether INH/LPS co-treatment with CYP2E1 inhibitor diallyl sulfide (DAS) and DEX can protect against the INH/LPS-induced hepatotoxicity. Our results showed that pre-administration of both DAS and DEX caused significant reduction in serum TBA, TBil, and gamma-glutamyl transferase levels. Furthermore, the histopathological analysis showed that DAS and DEX could effectively reverse the liver lesions seen following INH/LPS treatment and protect against hepatic steatosis as indicated by absence of lipid accumulation. Pre-treatment with DAS alone could not completely block the CYP2E1 protein expression following INH/LPS treatment, as appeared in the immunoblotting and immunohistochemistry results. This is probably due to the fact that the combined enhancement activities of both INH and LPS on CYP2E1 protein expression levels might resist the blocking probabilities of DAS. In the meantime, addition of DEX to the DAS/INH/LPS combination caused a significant reduction in CYP2E1 protein expression as revealed by the immunoblotting and fading coloration in immunohistochemistry results. Thus, addition of DEX and DAS together caused strong protection against INH/LPS-induced hepatic damage. These findings reveal the potential therapeutic value of combining DAS and DEX with INH in TB management for reducing the potential risk and incidences of hepatotoxicity.

## Introduction

Tuberculosis (TB) existed throughout human history as an infectious deadliest communicable diseases that affected humankind, with still high mortality and disease burden (Ramappa and Aithal, 2013; Caulfield and Wengenack, 2016; Cervantes, 2016). Meanwhile, the discovery and development of isoniazid (INH) in the 1950s paved the way to an effective single and/or combined first-line anti-TB therapy (Zumla et al., 2014). Since its introduction, INH remains one of the most irreplaceable antimicrobial medications with high bactericidal activity, high bioavailability, and excellent intracellular penetration (Whitney and Wainberg, 2002). Nevertheless, clinical utilization of INH is adversely associated with hepatotoxicity in which INH and its metabolites have been involved in hepatic toxicity in humans (Singh et al., 2011). INH-induced hepatotoxicity is a serious problem and mainly linked with therapy interruption and changes in treatment regimen during the TB management course (Tostmann et al., 2008; Khalili et al., 2009). The exact incidence of INH-induced liver injury is difficult to estimate retrospectively, mainly due to notorious cases underreporting and the contribution of co-medications. However, in their recent comprehensive prospective studies that included patients from different countries, many researchers have revealed that incidence of serious INH-induced liver has been in the range of 1–3% of treated (exposed) patients (Steele et al., 1991; Bjornsson et al., 2013; Shu et al., 2013).

As we previously described that one reason for the struggles in identifying susceptibility factors for hepatotoxicity in patients receiving anti-tubercular medications (mainly INH) is the lack of a clear understanding of the molecular mechanisms underlying INH-induced liver injury (Hassan et al., 2015). Despite these difficulties, our previous works (Hassan et al., 2016, 2017) achieved successfulness in establishing a 2-week INH hepatotoxic animal model implementing the inflammatory stress theory. In this model, implementation of endotoxic bacterial lipopolysaccharide (LPS), which are components from Gram-negative bacterial outer cell wall’s membrane, stimulates inflammatory response. As previously described by Andonegui et al. (2003), LPS is considered as a potent inflammagen and contributes significantly to the pathogenesis of Gram-negative bacterial infections by activating toll-like receptors on inflammatory cells, which in turn precipitate the expression of numerous soluble inflammatory mediators (Andonegui et al., 2003). Many preceding studies on LPS stated that exposure to large amounts of LPS during conditions such as sepsis is associated with fever, circulatory shock, disseminated intravascular coagulation, and injury to several organs, including the liver (Luyendyk et al., 2002; Neyrinck et al., 2007; Kozlov et al., 2009; Szabo and Csak, 2012). In contrast, another research outlined that small doses of LPS do not cause overt tissue injury but can nevertheless lead to generation, activation and tissue accumulation of inflammatory cells, and release of inflammatory mediators and pro-inflammatory cytokines and chemokines (Tomlinson and Blikslager, 2004). In the meantime, prior investigations in humans revealed that episodes of modest inflammation, although benign on their own, are probably commonplace in people and have the ability to augment the toxicities of several xenobiotic agents (Liu et al., 2000; Lu et al., 2011). Moreover, these inflammatory episodes occur irregularly and often go unnoticed, which have been led to the inflammatory stress hypothesis which speculates that an episode of inflammation concomitantly occurs during drug therapy might decrease the threshold for drug toxicity and thereby render an individual susceptible to an adverse drug reaction mainly hepatic toxicity (Roth et al., 1997; Deng et al., 2009). Using this *in vivo* inflammatory model may clarify mechanisms to minimize drug side effects or toxicities to determine if alternative drug candidates are likely to be safer. The ultimate goal will be to predict idiosyncratic reactions to early detect potential problems in drug discovery and development processes and could be used further to help develop and design suitable human clinical trials.

Previously, we highlighted the potential enhancement activities of LPS on INH-induced hepatic toxicity. These enhancement activities ranged between augmenting the inflammatory stress, oxidative stress, alteration of bile acid homeostasis, elevated cytochrome P450 2E1 (CYP2E1) expression, and both macro- and micro-steatosis (Hassan et al., 2016). Although pre-treatment with dexamethasone (DEX), a synthetic glucocorticoid that acts as a potent anti-inflammatory and immunosuppressive agent, helped overcome both inflammatory and oxidative stress which ended-up in alleviation of LPS augmenting effects, but still minor toxicities still being detected, alongside with CYP2E1 over expression (Hassan et al., 2017). This finding positively indicated the corner-stone role played by CYP2E1 in the pathogenesis of INH/LPS-induced liver damage. CYP2E1 is expressed in different organs including, lung and kidney as well as in the central nervous system of rats and humans, but the highest expression occurs in the liver (Hansson et al., 1990). Hepatic CYP2E1 is highly responsive to inflammatory inducers and mediators, such as LPS, which result in CYP2E1 significant repression (Abdel-Razzak et al., 1993; Kelicen and Tindberg, 2004). This repressive effect could be attributed to the reduction in HNF-1α, a transcription factor responsible for CYP2E1 down-regulation in response to inflammagens (Hakkola et al., 2003). From the other hand, our previous findings let us to speculate that CYP2E1 plays a cornerstone role in INH-induced hepatotoxicity that augmented by LPS (Hassan et al., 2016). Similarly, CYP2E1 was found to increase liver sensitivity toward LPS and inflammatory mediators’ toxicity, as well as, potentiates LPS-induced oxidative stress in the liver (Lu and Cederbaum, 2010; Cederbaum et al., 2012), while INH has also been shown to elevate CYP2E1 level (Yue et al., 2004; Lu and Cederbaum, 2008).

Garlic is a frequently used dietary herb. It is a commonly used foodstuff and possesses volatile oil that contains active principles such as diallyl sulfide (DAS), which acts as a potential biological antioxidant and a compelling free radical scavenger in suppressing the actions of reactive oxygen substances, thereby protecting the biomolecules against oxidative damage (Kalayarasan et al., 2008). DAS has attracted particular interest as a potential therapeutic or prophylactic agent because of its inhibitory actions on CYP2E1-mediated metabolic activation of various chemicals and carcinogens (Jin and Baillie, 1997). Organosulfur compounds present in garlic, including DAS, are capable of inhibiting CYP2E1 activity and expression (Davenport and Wargovich, 2005). Therefore, our main goals of the present study are to investigate and detect potential mechanisms behind CYP2E1 potential role in the INH/LPS hepatotoxicity model through CYP2E1 inhibition by DAS, and also DEX anti-inflammatory effects that further prevent the INH/LPS-induced CYP2E1 potentiation.

## Materials and Methods

### Drugs and Chemicals

Isoniazid (Lot No. MKBV9475V, analytical standard ≥ 99%), LPS (Lot No. 025M4128V, derived from *Escherichia coli* 0128:B12 serotype, source strain is CDC 2440-69), and DAS (Lot No. W204218, analytical standard ≥ 97%) were purchased from Sigma–Aldrich (St. Louis, MO, United States). DEX as sodium phosphate ready-made injections (Lot No. 5160303) was obtained from Hubei Chang Tian Pharmaceutical Company (Hubei, China).

### Reagents

Blood biochemical detection reagents were equipment-related reagents. Pathological sections-related reagents are provided by Pathology Department, Jiangsu Province Hospital of Integrated Traditional and Western Medicine. Trizol reagent was bought from Invitrogen Life Technologies (Carlsbad, CA, United States) while PrimeScript^@^RT Master Mix from Takara Biotechnology (Dalian, China) and SYBRGreen Supermix from Bio-Rad (Hercules, CA, United States). Other reagents were commercially available and of high analytical grade.

### Experimental Animals

Male, Sprague-Dawley (SD) rats weighing 200–220 g (age 8–12 weeks) were obtained from Shanghai Lingchang Biological Technology Co., Ltd. (Shanghai, China). All experimental procedures were conducted in accordance with the guide for Institutional Animal Care and Use Committee at China Pharmaceutical University and the National Institute of Health (NIH) guidelines for the care and use of laboratory animals. Rats were housed in controlled environmental conditions (24 ± 1°C, 52 ± 5% relative humidity, 12 h light–dark cycle) with free access to food and water *ad libitum*. Animals were acclimatized for 1 week before conducting the experiments. All experimental procedures were approved by China Pharmaceutical University, National Drug Screening Center, and Jiangsu Institute of Materia Medica Ethical Committees, Nanjing, China.

### Experimental Design and Drugs Regimen

Our previous experimental data revealed that INH in a concentration of 400 mg/kg is associated with high incidence of liver toxicity (Su et al., 2014; Hassan et al., 2016, 2017). Therefore, in this study, we choose this dose in order to verify the role of CYP2E1 in INH/LPS-induced liver injury.

Adult male SD rats (*n* = 7 per group) were randomly divided into six groups: control group (group I), INH 400 mg/kg (i.g.) plus intravenous LPS 2 mg/kg (group II), DAS 200 mg/kg plus INH 400 mg/kg (i.g.) plus intravenous LPS 2 mg/kg (group III), DAS 200 mg/kg plus INH 400 mg/kg (i.g.) plus intraperitoneal DEX 4 mg/kg plus intravenous LPS 2 mg/kg (group IV) while both group V and group VI received the same intragastric DAS and intraperitoneal DEX doses, respectively.

Diallyl sulfide was intragastrically administered, starting from 3 days before conducting the experiment, and daily continued throughout the entire experimental period. INH was intragastrically administered for 14 consecutive days 1 h after DAS, whereas LPS was given as intravenous bolus dose at day 14, 2 h before the last INH dose. DEX was also given as single intraperitoneal dose at day 14, 1 h prior LPS administration. These selected concentrations were in accordance with previous research on DEX (Dandona et al., 1999; Eum et al., 2003; Cui et al., 2015) and DAS (Brady et al., 1988; Yang et al., 2001). Rats were sacrificed after INH last dose; blood was collected, allowed to clot at room temperature, and centrifuged at 3500 rpm for 10 min for serum collection. Liver sections were isolated, frozen in liquid nitrogen, and then stored for further experiments.

### Serum and Liver Biochemical Index

Immediately following blood collection and centrifugation at 3500 rpm for 10 min, the supernatant serum was used for hepatotoxicity determination, following the standard enzymatic techniques. Serum alanine transaminase (ALT), aspartate transaminase (AST), total bile acids (TBA), total bilirubin (TBil), gamma-glutamyl transferase (γGGT), triglyceride (TG), and total cholesterol (TC) levels were measured by HITAC7170A automatic analyzer (Hitachi, Japan) in accordance with standard spectrophotometric methods. Both liver TG and TC were determined following the manufacturer’s instructions of corresponding detection kits (Nanjing Jiancheng Bioengineering Institute, Nanjing, China). These indices reflect both degree of liver cell’s damage (ALT and AST) and liver secretion and excretion (TBA, TBil, and γGGT), while lipid metabolic function reflected by TC and TG.

### Histopathology Study

After tissue isolation, livers were visually inspected for any color or texture abnormalities, and subsequently washed with 0.9% normal saline, dried, and weighed. Histopathology analyses were conducted on rat livers slices, in which they were fixed in 10% paraformaldehyde solution and embedded in paraffin wax. Sections were cut at 4–5 μm thickness and stained with hematoxylin–eosin (HE) staining. Slides were coded, randomized, and evaluated by pathophysiologists who were ignorant to the treatment schedule. Then after, slides were photographed using the 1X81 Olympus confocal laser scanning microscope (Olympus, Japan).

### Oil Red O Staining

To further evaluate hepatic steatosis following INH/LPS co-treatment, frozen liver samples were sliced and then stained for lipids using standard Oil Red O protocol at Jiangsu Traditional Chinese Combined with Western Medicine Hospital (Nanjing, China).

### Measurement of Antioxidant Enzymes and MDA Levels

A total of 0.1 g of liver tissue sample from all groups were homogenized in normal saline (HerosMole TL2010S Homogenizer, Beijing, China) and subjected to analysis for detection of superoxide dismutase (SOD), reduced glutathione (GSH), malondialdehyde (MDA), and liver total antioxidant capacity (T-AOC) through their corresponding assays which carried out using appropriate kits (Nanjing Jiancheng Bioengineering Institute, Nanjing, China), in accordance with the manufacturer’s instructions.

### Quantitative Real-Time Polymerase Chain Reaction (qPCR)

Briefly, total RNA was extracted from rat’s liver tissues by Trizol reagent (Invitrogen Life Technologies, Carlsbad, CA, United States) following manufacturer’s instructions provided and quantified using 2000 Nanodrop Spectrophotometer (Thermo DE, United States). Then after, 2 μg of total RNA of each sample was then reverse-transcribed into complementary DNA (cDNA) using PrimeScript@ RT Master Mix (Takara Biotechnology, Dalian, China), following the manufacturer’s instructions. The qPCR was executed in 20 μl volume containing 10 μl SYBRGreen Supermix (Bio-Rad, Hercules, CA, United States), 1 μl of cDNA, 7 μl of RNase-/DNase-free water, and 500 nM each primer. The incorporation of the SYBRGreen dye into the PCR products was monitored in real-time using the iCycler iQ5 Multicolor Real-Time PCR Detection System (Bio-Rad, Hercules, CA, United States), and the resulting threshold cycle (*C*_t_) values were computed. The thermal cycler conditions were as follows: 30 s at 95°C, followed by 40 cycles of 5 s at 95°C and 10 s at 60°C. A melting curve analysis was carried out for each reaction from 65 to 95°C. The threshold cycle at which the fluorescent signal reached an arbitrarily set threshold near the middle of the log-linear phase of amplification for each reaction was calculated, and relative quantities of each mRNA were determined. All measurements were done in three independent replicates and additionally three replications within each qRT-PCR run. Values, expressed as the relative mRNA level of specific target gene, were normalized to glyceraldehyde-3-phosphate dehydrogenase (GAPDH) gene levels that served as a reference, as obtained using the ΔΔ*C*_T_ method (Livak and Schmittgen, 2001). The specific primers used in this study were synthesized, purified, and quality-inspected by Invitrogen Biotechnology (Shanghai, China) and their sequences were listed in Supplementary Table S1.

### Protein Extraction and Western Immunoblotting

For liver total protein extraction, 100 mg of liver samples was homogenized with radioimmunoprecipitation assay (RIPA) buffer and phosphatase and protease inhibitors (Vazyme Biotech, Nanjing, China) following the manufacturer’s instructions. Total protein concentrations were determined by the bicinchoninic acid (BCA) method. Protein extracts were mixed with Laemmli loading buffer at 1:4 ratio (v/v), boiled at 100°C for 5 min, and stored at -20°C until use. For western blot analysis, equal amounts of proteins (70 μg) were electrophoresed on sodium dodecyl sulfate–polyacrylamide gel electrophoresis (SDS–PAGE); separated proteins were transferred to polyvinylidene difluoride (PVDF) membranes (Bio-Rad, Hercules, CA, United States). After nonspecific blocking with 5% skim milk for 1 h, the membranes were probed with primary antibody (1:500 to 1:1,000) in 5 ml blocking buffer overnight at 4°C, then washed with Tris-buffered saline with Tween 20 (TBST) buffer, and incubated with suitable horseradish peroxidase (HRP)-conjugated secondary antibody. Membranes were further washed with TBST, incubated with an ECL solution (Millipore, United States), and digitally imaged with a charge-coupled device (CCD) camera. All antibodies utilized in this study were listed in Supplementary Table S2. All experiments were performed three times and relative protein expressions were compared to β-actin expression that set as control.

### Terminal Deoxynucleotidyl Transferase dUTP Nick-End Labeling (TUNEL) Staining and Immunohistochemistry

Paraffin-embedded liver sections were stained by TUNEL assay in order to identify apoptotic hepatocytes using TUNEL Detection Kit (KeyGen Biotech, Nanjing, China) following the manufacturer’s guidelines. TUNEL-stained liver samples were captured with light microscope (Olympus IX81, Japan). Immunohistochemistry testing was performed for CYP2E1 using corresponding antibodies on hepatic tissues fixed in formaldehyde and embedded in paraffin slices. In brief, liver sections were fixed in formalin and permeabilized using 0.1% Triton X-100. Sections were pre-treated with 0.3% BSA to block nonspecific binding and incubated with rabbit antibody against CYP2E1 overnight, following the treatment with Alexa Fluor 594 Donkey anti-Rabbit lgG Antibody, provided by Invitrogen Life Science (Carlsbad, CA, United States). CYP2E1 immunostaining images were captured by Olympus IX81 Motorized Inverted Fluorescence Microscope.

### Statistical Analysis

Experimental data were presented as means ± SD and analyzed using GraphPad Prism 6 (GraphPad Software, Inc., San Diego, CA, United States). Statistical comparisons between both treated and untreated group were performed by one-way analysis of variance (ANOVA), difference between two groups was analyzed by Student’s two-tailed *t*-test. For all analyses, *P*-value <0.05 was considered statistically significant.

## Results

### DAS and DEX Co-administration Protects Against INH/LPS-Induced Hepatotoxicity

**FIGURE 1:**
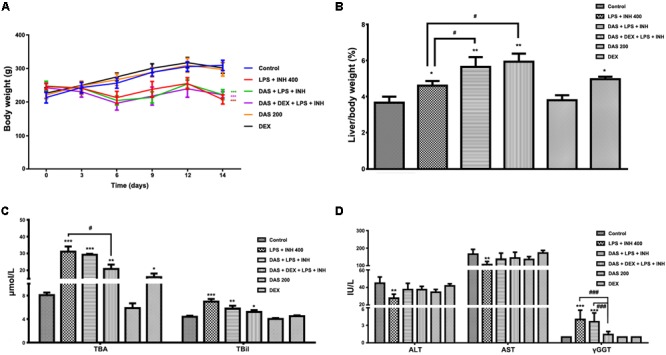
Effects of DAS and DEX administration on liver injury parameters induced by INH/LPS co-administration. SD rats were intragastrically treated with DAS (200 mg/kg) starting from 3 days before INH administration and daily continued throughout the experimental period. INH 400 mg/kg was intragastrically administered for 14 consecutive days 1 h after DAS, whereas LPS (2 mg/kg) was given as intravenous bolus dose at day 14, 2 h before the last INH dose. DEX (4 mg/kg) was also given as single intraperitoneal dose at day 14, 1 h prior LPS administration. **(A)** Alteration in body weight. **(B)** Liver coefficient after drugs treatment. **(C)** Impact on serum TBA and TBil levels. **(D)** Alterations in serum ALT, AST, and γGGT levels. Data are presented as mean ± SD, *n* = 7 for each bar, ^∗^*P* < 0.05, ^∗∗^*P* < 0.01, ^∗∗∗^*P* < 0.001 versus control, ^#^*P* < 0.05, ^##^*P* < 0.01, ^###^*P* < 0.001 versus INH/LPS or DAS/INH/LPS combination.

Animals that received INH/LPS combined with either DAS alone or both DAS and DEX showed significant reduction in their body weights (*P* < 0.001) compared to the control group. In the meantime, rats administered DAS or DEX alone showed insignificant difference in their body weight compared to the control group. Meanwhile, liver coefficient analysis revealed significant increment (*P* < 0.05) in rats receiving combination of INH and LPS, whereas addition of DAS and DEX caused significant elevation in liver coefficient (*P* < 0.01), which could be ascribed to reduction in total body weights compared to the liver weights (**Figures 1A,B**). In the meantime, serum ALT and AST levels still significantly reduced (*P* < 0.01) in rats received INH/LPS combination, whereas pre-addition of both DAS and DEX to INH/LPS co-treated animals raised both ALT and AST levels, but still beneath control level (**Figure 1C**). Furthermore, serum TBA, TBil, and γGGT were significantly raised (*P* < 0.001) following INH/LPS treatment, whereas pre-addition of DAS alone caused insignificant changes in their levels compared to both control and INH/LPS groups, while pre-administration of both DAS and DEX caused marked reduction in their serum levels, which were almost indifferent from the control group levels (**Figures 1C,D**).

### Histopathological Examination Analysis of Liver Tissues

**FIGURE 2:**
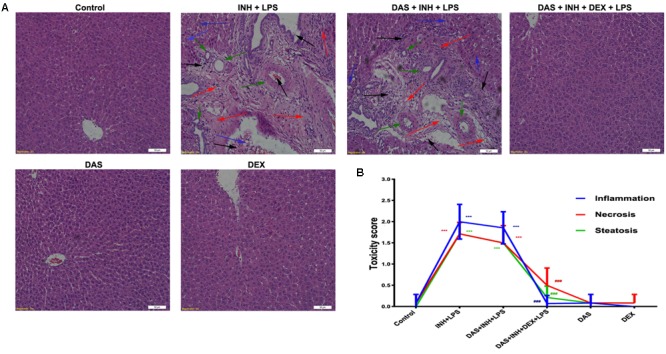
Liver histopathological analysis after 2-week INH/LPS co-treatment combined with DAS and DEX. **(A)** Liver slices were collected and subjected to staining with hematoxylin and eosin. The control group shows normal hepatocyte architecture; meanwhile, INH/LPS co-treated animals’ liver showed sever toxicity symptoms. Inflammatory cells and inflammatory infiltration (black arrow), bile duct hyperplasia (green arrows), micro- and macrovesicular steatosis (blue arrow), massive necrosis, and hepatocellular structure loss (red arrow). Furthermore, addition of DAS revealed the same toxicity symptoms. On the other hand, both DAS and DEX pre-treatment caused complete liver protection as appeared by the absence of these liver injury indicators. **(B)** INH/LPS hepatotoxicity score in the absence or presence of DAS and DEX. Data are given as means ± SD, *n* = 7 for each bar, ^∗∗∗^*P* < 0.001 versus control, ^###^*P* < 0.001 versus INH/LPS combination.

Histopathological examination of liver sections showed that control, DAS-alone, and DEX-alone groups had a normal lobular architecture with normal cell morphology and no obvious pathological state, whereas severe and extensive damage was seen in INH/LPS-treated rats (**Figure 2A**). The features of this massive damage include intense micro- and macro-vesicular steatosis, severe hepatocellular necrosis, loose irregular arrangement of liver cells, and inflammatory infiltration. Furthermore, addition of DAS did not cause any protection or improvement, as the extensive damage features still being seen. From the other hand, DAS and DEX combination revealed complete protection against INH/LPS co-treatment-induced hepatotoxicity. These results were confirmed by the toxicity score analysis (**Figure 2B**).

### Effect on Hepatic Lipid Levels

**FIGURE 3:**
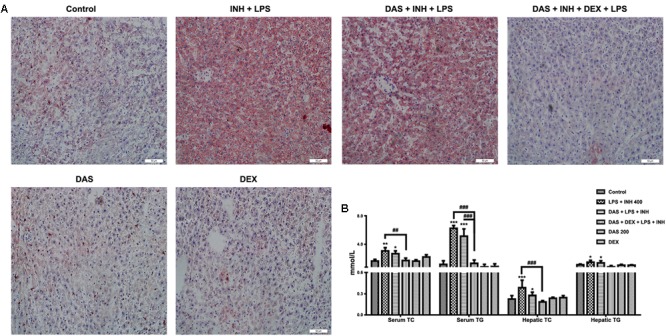
Analysis of lipid profile following drug treatment. **(A)** Lipid staining in rat livers. Frozen liver sections were subjected to Oil Red O staining; massive steatosis seen in rats received INH/LPS co-treatment with or without DAS administration, whereas combination of DAS and DEX decreased hepatic lipid accumulation. **(B)** Variation in both serum and hepatic lipid profile. Data are represented as mean ± SD, *n* = 7 for each bar. ^∗^*P* < 0.05, ^∗∗^*P* < 0.01, ^∗∗∗^*P* < 0.001 versus control, ^##^*P* < 0.01, ^###^*P* < 0.001 versus INH/LPS or DAS/INH/LPS combination.

Oil Red O staining was carried out to verify the presence of steatosis, as previously indicated in the histopathological analysis. As shown in **Figure 3A**, 400 mg/kg INH plus LPS with or without DAS animal groups displayed intense micro- and macro-vesicular steatosis as confirmed by the strong staining, while liver slides from DEX pre-treated rats revealed absence of staining indicating no accumulation of lipid droplets, implying that DEX minimizes the steatotic-induction ability of INH/LPS co-treatment. Further validation of this anti-steatotic ability seen with DEX, both hepatic TG and TC were quantified using their corresponding quantification kits. Hepatic TC level was significantly increased (*P* < 0.001) in INH/LPS-treated animals (**Figure 3B**). Meanwhile, hepatic TG level also showed significant elevation (*P* < 0.05) in rats administered INH/LPS combination. Addition of DAS alone revealed minor differences compared to the results already noticed with INH/LPS combination, but in rats pre-treated with both DAS and DEX, their liver TG levels rendered almost as same as normal control levels. Moreover, serum TC and TG levels significantly elevated (*P* < 0.01 and *P* < 0.001, respectively) after administration of INH/LPS combination, which were significantly reduced following DEX and DAS co-treatment (**Figure 3B**).

### Evaluation of Oxidative Stress Markers

**FIGURE 4:**
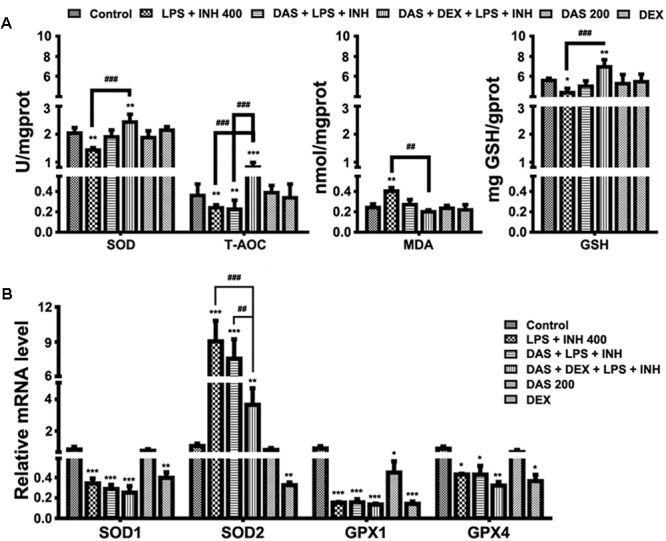
Variations in oxidative stress and hepatic antioxidant defense mechanisms. **(A)** Changes in SOD, T-AOC, MDA, and GSH levels were measured by their respective kits. **(B)** Antioxidant gene expressions after drugs administration. Data are represented as mean ± SD, *n* = 7 for each bar. ^∗^*P* < 0.05, ^∗∗^*P* < 0.01, ^∗∗∗^*P* < 0.001 versus control, ^##^*P* < 0.01, ^###^*P* < 0.001 versus INH/LPS or DAS/INH/LPS combination, GAPDH was set as reference control gene.

It is important to recognize that oxygen activation by P450, necessary for the enzyme’s catalytic function, can also result in the production of reactive oxygen species (ROS) (White, 1991; Loida and Sligar, 1993). Considering the key roles of ROS in the development of INH/LPS-induced hepatotoxicity, it was necessary and important to investigate the changes of the hepatic antioxidant system, which were displayed in **Figure 4**. As shown in **Figure 4A**, we found that the INH/LPS combination significantly decreased (*P* < 0.01) both the hepatic SOD and T-AOC levels, while addition of DAS only reversed the SOD level to that observed in the control group with no effect seen on T-AOC level. On the other hand, DAS and DEX co-administration significantly elevated both SOD and T-AOC levels (*P* < 0.01 and *P* < 0.001, respectively). Meanwhile, analysis of MDA, as one of the major lipid peroxidation parameters, showed significant elevation (*P* < 0.01) in INH/LPS-co-treated rats, whereas additions of DAS and DEX, either alone or in combination, led to restoration of their MDA levels to the normal control ones. For hepatic GSH, the major intracellular antioxidant defense mechanism, liver GSH level exhibited significant inhibition in INH/LPS (*P* < 0.05). Meanwhile, rats pre-treated with DAS and DEX revealed improvement in GSH levels, with significant elevation in rats administered DAS/DEX combination. These results indicated that hepatic antioxidant abilities have been raised due to the DAS/DEX pretreatment which led to protection against INH/LPS-induced liver damage.

To confirm the role of DAS/DEX in improving hepatic antioxidant mechanisms, we further analyzed gene expressions of SOD1, SOD2, glutathione peroxidase 1 (GPX1), and GPX4 which functions in the detoxification of oxygen-free radicals. Except for SOD2, all other genes showed significant reductions (*P* < 0.001) in groups received INH/LPS combined with DAS or DAS/DEX. Meanwhile, SOD2 showed significant over expression (*P* < 0.001) in INH/LPS and DAS with INH/LPS groups (**Figure 4B**).

### DAS and/or DEX Effects on Selected Genes Profile Associated With INH/LPS-Induced Hepatotoxicity

**FIGURE 5:**
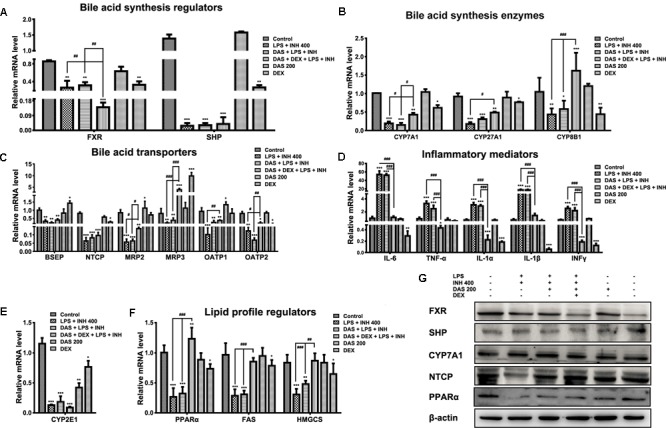
Changes in different targets’ genes and proteins expressions following INH/LPS treatment with or without DAS and DEX. **(A)** Bile acid regulators FXR and SHP expression. **(B)** Expression of bile acid synthesis enzymes CYP7A1, CYP27A1, and CYP8B1. **(C)** Bile acid transporters BSEP, NTCP, MRP2, MRP3, OATP1, and OATP2 expression. **(D)** Inflammatory mediators IL-6, TNFα, IL-1α, IL-1β, and INFγ expression. **(E)** Expression of CYP2E1. **(F)** Lipid profile regulators PPARα, FAS, and HMGCS expression. Data were represented as mean ± SD, *n* = 7 for each bar. ^∗^*P* < 0.05, ^∗∗^*P* < 0.01, ^∗∗∗^*P* < 0.001 versus control, ^#^*P* < 0.05, ^##^*P* < 0.01, ^###^*P* < 0.001 versus INH/LPS or DAS/INH/LPS combination, GAPDH was set as reference control gene. **(G)** Immunoblotting analysis of different targeted proteins following INH/LPS with or without DAS and DEX treatment, β-actin considered as loading control.

The gene expression profiles associated with INH/LPS hepatotoxicity with or without pre-administration of DAS and DEX were displayed in **Figure 5**. As we suggested before, massive accumulation of bile acid in the hepatocytes could be one of the major pathways behind INH/LPS-induced severe liver damage which further support the overall reduction seen in bile acid-related genes (Hassan et al., 2016, 2017), in this part we extend our focusing to the effects of both DAS and DEX on those gene expressions. As shown in **Figure 5A**, the expression levels of the orphan nuclear receptor farnesoid X receptor (FXR) and the small heterodimer partner (SHP) were significantly repressed (*P* < 0.01 and *P* < 0.001, respectively). Interestingly, pre-addition of both DAS and DEX didn’t restore the expression of these genes to their corresponding control levels. Furthermore, expressions of bile acids synthesizing genes, namely CYP7A1, CYP27A1, and CYP8B1, had been significantly repressed following INH/LPS treatment (*P* < 0.001), whereas pre-administration of DAS alone didn’t modify these gene expressions (**Figure 5B**).

Meanwhile, combination of DAS and DEX helped elevate the expressions of CYP7A1 and CYP27A1 which were still beneath their corresponding control group levels, while CYP8B1 showed significant over-expression (*P* < 0.001) compared to both control and drugs-treated groups. Moreover, we study the effects of DAS and DEX on the bile acid transporters BSEP, NTCP, MRP2, MRP3, OATP1, and OATP2 (**Figure 5C**) in which INH/LPS significantly repressed these transporters’ expressions, while DAS pre-addition showed no signs of any improvement except for OATP1 and OATP2, but both DAS and DEX caused significant over-expression of MRP3 transporter (*P* < 0.001), whereas changes on the other transporters were significant in term of elevation compared to the INH/LPS group. These transporters repression seen following INH/LPS treatment is associated with the elevated serum TBAs level and accumulation of toxic bile acid which prompted hepatocyte necrosis.

For the inflammatory mediator expressions, it was clearly observed that DAS is completely lacking any anti-inflammatory activities, as indicated by the overexpression of the major inflammatory cytokines IL-6, TNF-α, IL-1α, IL-1β, and INFγ (**Figure 5D**). In the meantime, DEX, through its potent anti-inflammatory activities, significantly prevented the expression of the inflammatory cytokines (*P* < 0.001). Meanwhile, CYP2E1 expressions were significantly reduced following drugs treatment (**Figure 5E**).

Histopathological and Oil Red O staining results were associated with severe micro- and macro-vesicular steatosis in groups received INH/LPS with or without DAS, while addition of DEX protects against INH/LPS-induced hepatic steatosis. Furthermore, serum and liver lipid parameters were consistent with these findings in which both TC and TG were elevated. Accordingly, analysis of lipid profile regulators, namely PPARα, fatty acid synthase (FAS), and hydroxymethylglutaryl-CoA synthase (HMGCS), showed significant reductions (*P* < 0.001) which explained the massive accumulation of lipids seen in INH/LPS with or without DAS. Interestingly, as previously observed, addition of DEX caused significant PPARα overexpression (*P* < 0.01) and returned the levels of lipid synthesizing enzymes toward their corresponding control levels (**Figure 5F**).

### Results of Western Immunoblotting

Analysis of western immunoblotting for selected parameters was carried out in order to confirm our results. As shown in **Figure 5G**, both FXR and SHP protein levels were significantly reduced in INH/LPS with or without DAS and DEX; meanwhile, CYP7A1 expression was higher in INH/LPS groups, even when DAS was pre-administered, whereas addition of DEX clearly reduced the CYP7A1 protein level. Meanwhile, NTCP expression was blocked by INH/LPS; however, addition of either DAS alone or DAS and DEX combination resulted in NTCP overexpression. From the other hand, PPARα followed the same behavior of its gene expression, in which INH/LPS caused severe inhibition at its protein level which was not fully improved by DAS pre-treatment, but its expression was up-regulated in the DEX pre-administered groups.

### The Potential Role of CYP2E1 in INH/LPS Hepatotoxicity

**FIGURE 6:**
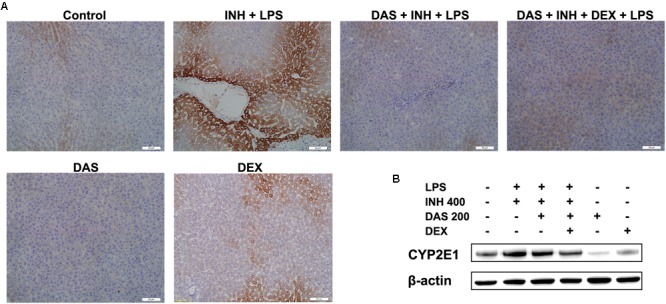
Hepatic CYP2E1 protein expression levels following drugs administration. **(A)** INH/LPS treatment elevated CYP2E1 expression (deep brown color). Moreover, DAS-only administration to the animals received INH/LPS marginally reduced CYP2E1 expression, but addition of DEX to the combination significantly inhibited CYP2E1 protein expression probably through elimination of LPS-enhancing effects, as indicated by the fading color. **(B)** CYP2E1 immunoblotting analysis, β-actin used as internal control.

In our previous studies, we came in a conclusion that both INH and LPS either individually or combined together have the potentiality to elevate the CYP2E1 protein expression, and DEX alone only reduced the LPS-enhancement abilities on CYP2E1 levels. In the current study, following treatment with INH/LPS either alone or combined with DAS and DEX, in an opposite to their effects on CYP2E1 mRNA level, INH/LPS provoked the CYP2E1 protein expression as indicated by both immunoblotting and immunohistochemistry (strong brown color indicating high levels of CYP2E1). Pre-treatment with DAS alone could not completely block the CYP2E1 protein expression following INH/LPS treatment, as appeared in the immunoblotting and immunohistochemistry results, although groups that received DAS alone throughout the experimental duration showed successive complete CYP2E1 inhibition as indicated in **Figures 6A,B**. This is probably due to the fact that the combined enhancement activities of both INH and LPS on CYP2E1 protein expression levels might resist the blocking probabilities of DAS. In the meantime, addition of DEX to the DAS/INH/LPS combination caused a significant reduction in CYP2E1 protein expression as revealed by the immunoblotting and fading coloration in immunohistochemistry results.

### Hepatocellular Apoptosis as a Major Pathway in INH/LPS-Induced Hepatotoxicity

**FIGURE 7:**
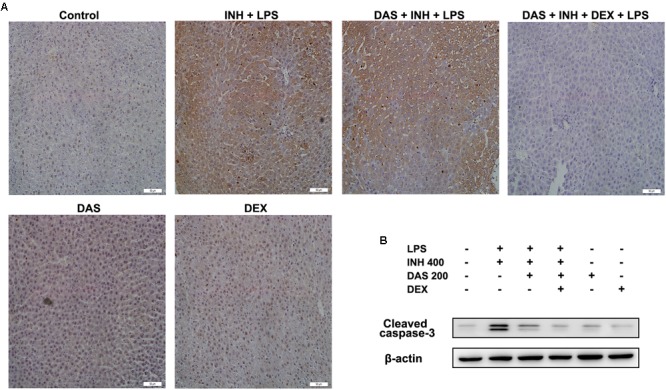
Hepatocellular apoptosis following INH/LPS treatment with or without pre-addition of DAS and DEX. **(A)** TUNEL assay was conducted on liver slices; INH/LPS co-administration with or without DAS caused obvious positive TUNEL results (intensity of brown color indicating apoptotic action). In the meantime, pre-addition of DEX restrained INH/LPS tendency to induce apoptosis (absence of brown color). **(B)** Representative western blot analysis of cleaved caspase-3, β-actin used as internal control.

Apoptosis is considered as one of the principal cellular death mode in injured liver, which could be easily detected through TUNEL assay (Wang, 2014). As demonstrated in **Figure 7A**, a positive TUNEL result seen in INH/LPS-treated animals either with or without pre-administration of DAS. Although DAS alone failed to protect hepatocellular apoptotic effects induced by INH/LPS co-treatment, addition of DEX caused valuable protection against apoptosis. Further verification has been done through measuring the protein expression of hepatic cleavage caspase-3, as a key indicator of cellular apoptosis. A high protein levels were measured in animals received INH/LPS combination, while still noticeable levels of the protein detected in animals treated with INH/LPS in addition to DAS only. Meanwhile, DEX addition revealed no levels of cleaved caspase-3 (**Figure 7B**).

## Discussion

In this current study, we investigated the potential role of CYP2E1 in the mechanisms of INH/LPS-induced hepatotoxicity through pre-administration of DAS, a potent CYP2E1 inhibitor. Our previous work indicated that addition of DEX, a potent anti-inflammatory agent, although blocked the enhancement effects of LPS on INH hepatotoxicity, but it didn’t completely protects against liver injury caused by the INH/LPS combination, which we attributed to the CYP2E1. Hence, we assumed that blocking of both inflammation and CYP2E1 will result in total protection against INH/LPS-induced hepatic toxicity. DAS is an organosulfur component of garlic, very well known for its antioxidant property (Grudzinski et al., 2001) and anti-inflammation activity (Kalayarasan et al., 2009).

Previously, we concluded that substitution of the classical hepatotoxicity parameters (ALT and AST), with other blood biochemical indices (mainly TBA, TBil, and γGGT) would be beneficial in order to positively detect INH hepatotoxicity, in which both ALT and AST considered to be time-dependent, their level elevates with longer drug-contact time (Karthikeyan, 2004; Metushi et al., 2012; Jahan et al., 2015; Hassan et al., 2016, 2017), therefore will not give the exact incidence of liver injury. In this recent experiment, serum ALT and AST levels showed the same reductions previously occurred following INH/LPS administration, whereas pre-treatment with DAS and DEX maintained their levels near normal. This protection was confirmed by the histopathological findings, where pre-addition of DAS-alone to the INH/LPS combination failed to protect the liver injury seen, which could be attributed to the synergistic effects of LPS on INH, while combination of both DAS and DEX completely prevent the hepatic toxicity. Failure of DAS-alone to maintain full protection over INH/LPS toxicity might be due to the inflammatory stress generated by LPS. Previous report also positively linked between garlic and stimulation of inflammatory cytokines produced by LPS (Sung et al., 2015). This observation was also noticed with the serum bile acid parameters and the expression levels of bile acid synthesis and transport-associated genes. It is clearly appeared from the results that DAS-alone only slightly reduced the serum TBA, TBil, and γGGT levels, which could be attributed to the blocking of CYP2E1 in which the later also had a functional role in bile acid increment through activation of different components in the bile acid synthesis cascade (Cheng et al., 2013). Addition of both DAS and DEX to INH/LPS combination protect against accumulation of hepatocellular toxic bile acids, as indicated by the repression of bile acids direct synthesizing enzymes (CYP7A1 and CYP27A1), while CYP8B1 mRNA overexpression might also prove the reduction in bile acid levels (Fan et al., 2014). Furthermore, DEX enhancing effects on MRP3 expression also protects against accumulation of intracellular toxic bile acids (Mörk et al., 2016).

Positive links between INH, inflammation by endotoxic LPS, and CYP2E1 already have been established, as both INH and LPS provoked CYP2E1 expression, which in return increases the sensitivity of body organs, including the liver, to their potential toxicities (Yue et al., 2004; Cheng et al., 2013; Shayakhmetova et al., 2015). Moreover, CYP2E1-induced oxidative stress and ROS production have been directly associated with liver damage (Rendic, 1999; Ramaiah et al., 2001; Abdelmegeed et al., 2012). Studies have been already reported that in healthy animals, there is an antioxidant defense system against ROS-mediated cell damage represented by endogenous antioxidants such as GSH and enzymatic scavengers such as SOD and catalase (CAT), these antioxidants neutralize, metabolize, or remove free radicals and protect the cells against oxidative damage (Abdel-Daim et al., 2014, 2015). DAS is an efficient free radical scavenger (Heo et al., 2007) and is well-known for its antioxidant properties (Yin et al., 2002; Hassan et al., 2010; Abdel-Daim and Abdou, 2015). Furthermore, early researches already summarized that DAS and other related compounds have been shown to suppress oxidative stress-induced tissue damage by increasing SOD and GSH activities and decreasing MDA levels (Brady et al., 1991; Pedraza-Chaverri et al., 2003a,b; Iranloye and Oludare, 2011; Ho et al., 2012). These facts were in line with our results, in which addition of DAS-alone marginally increased GSH and SOD and reduced MDA levels, but didn’t fully increase the hepatic antioxidant defense mechanism which could be due to the mimicking capacities of both CYP2E1 and LPS (Xu et al., 2017). Further addition of DEX significantly elevated GSH and SOD levels while severely reduced MDA level and increased the hepatic T-AOC. Taken together, blocking of both CYP2E1 and LPS inflammation diminished the INH-induced ROS generation and elevated the overall hepatic antioxidant capabilities.

From analysis of our results, it is obviously cleared that DAS lacks any potent anti-inflammatory actions, as indicated by the accumulation of the inflammatory cytokine mediators. Although DAS as well as other sulfur-containing compounds in garlic have demonstrated positive anti-inflammatory effect (Lee et al., 2012), but these potent anti-inflammatory actions were diminished in the group that received DAS with INH/LPS. Possible reasons behind this might be the forceful inflammation that generated from INH, LPS, as well as accumulation of toxic bile acids levels. Moreover, DAS-alone didn’t protects the INH/LPS-induced hepatic steatosis as evident by the massive accumulation of lipid droplets and elevated both serum and hepatic lipid parameters, which further associated with repressions of lipid profile regulators PPARα, FAS, and HMGCS. This further fortified our primary hypothesis that the reduction in PPARα activity that occurred due to inflammatory stress will promote further inflammation and lipid metabolism disturbances, which explains the elevated level of inflammatory mediators and the accumulation of TGs in liver tissues (Hassan et al., 2016). In contrast to our results, anti-inflammatory effects of DAS were further highlighted in the study conducted with rat aortic smooth muscle A7r5 cells (Ho et al., 2014), in which pretreatment with DAS was shown to block TNF-α- and histamine-mediated inflammatory responses. Specifically, DAS pretreatment attenuated TNF-α-induced enhanced expression of TNF-α and IL-1β transcription in A7r5 cells. Moreover, DAS-mediated anti-inflammatory effects were also found to be effective in animal model studying bleomycin-induced pulmonary fibrosis (Kalayarasan et al., 2008), and the beneficial anti-inflammatory effects of DAS were also observed in chondrocytes and synovial cells of osteoarthritis patients (Lee et al., 2009).

**FIGURE 8:**
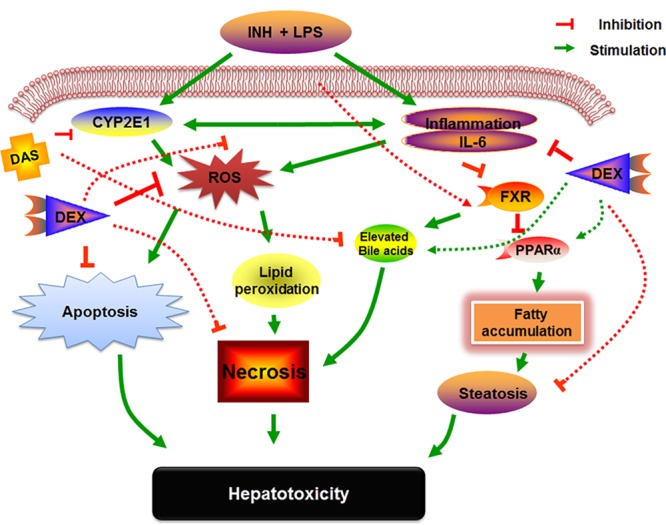
Schematic representation indicating the suggested mechanisms by which INH/LPS-induced hepatotoxicity and the DAS and DEX possible mechanistic intervention for liver protection in INH/LPS liver injury model.

Alongside it is a central key role in ROS generation, CYP2E1 was found to play an essential role in INH/LPS-induced hepatotoxicity. Documentations regarding participations of both INH and its metabolites in the induction of hepatic CYP2E1 are available (Yue et al., 2004; Cheng et al., 2013). Meanwhile, reports regarding the link between LPS and CYP2E1 revealed that both LPS and CYP2E1 were found to be sharing a unique relationship in which bacterial LPS not only stimulates CYP2E1 expression, but also increases the hepatic sensitivity toward CYP2E1 that further exaggerates INH/LPS toxicity (Lu and Cederbaum, 2010; Cederbaum et al., 2012; Shayakhmetova et al., 2015). DAS has attracted a particular interest as a potential therapeutic or prophylactic agent because of its inhibitory actions on CYP2E1-mediated metabolic activation of various chemicals and carcinogens. Organosulfur compounds present in garlic, including DAS, are capable of inhibiting CYP2E1 activity and expression (Rao et al., 2015). Interestingly, DAS didn’t completely blocked CYP2E1 expression following INH/LPS co-treatment, which again might be due to the dual stimulating activity of both INH and LPS. As DAS considered as a CYP2E1 competitive antagonist, it possibly failed to inhibit CYP2E1 expression that occurs as a consequence of both INH and LPS. This was further confirmed with the addition of DEX that suppresses the LPS-stimulating tendencies of CYP2E1. Our overall pondering of DAS and DEX pre-administration to INH/LPS-treated rats can be fully elucidated in **Figure 8**.

In summary, this study investigates the potential role of CYP2E1 in the pathogenesis behind INH/LPS-induced liver injury. Both INH and LPS have synergistic activity in CYP2E1 induction, as indicated by DAS incomplete CYP2E1 inhibition that caused minor protection against INH/LPS-induced liver injury. Addition of DEX and DAS together caused strong protection against INH/LPS-induced hepatic damage. These findings reveal the potential therapeutic value of combining DAS and DEX with INH in TB management for reducing the potential risk and incidences of hepatotoxicity.

## Author Contributions

HH, HG, ZJ, and LZ participated in the research design. HH, LX, and HG carried out the experiments. HH and BY performed the data analysis. HH and ZJ wrote the manuscript.

## Conflict of Interest Statement

The authors declare that the research was conducted in the absence of any commercial or financial relationships that could be construed as a potential conflict of interest.

## References

[B1] Abdel-Daim M. M. Abd Eldaim M. A. Mahmoud M. M. (2014). *Trigonella foenum-graecum* protection against deltamethrin-induced toxic effects on haematological, biochemical, and oxidative stress parameters in rats. *Can. J. Physiol. Pharmacol.* 92 679–685. 10.1139/cjpp-2014-0144 25029214

[B2] Abdel-Daim M. M. Abdou R. H. (2015). Protective effects of diallyl sulfide and curcumin separately against thallium-induced toxicity in rats. *Cell J.* 17 379–388. 26199917 10.22074/cellj.2016.3752 PMC4503852

[B3] Abdel-Daim M. M. Ghazy E. W. Fayez M. (2015). Synergistic protective role of mirazid (*Commiphora molmol*) and ascorbic acid against tilmicosin-induced cardiotoxicity in mice. *Can. J. Physiol. Pharmacol.* 93 45–51. 10.1139/cjpp-2014-0336 25429612

[B4] Abdelmegeed M. A. Banerjee A. Yoo S. H. Jang S. Gonzalez F. J. Song B. J. (2012). Critical role of cytochrome P450 2E1 (CYP2E1) in the development of high fat-induced non-alcoholic steatohepatitis. *J. Hepatol.* 57 860–866. 10.1016/j.jhep.2012.05.019 22668639 PMC3445664

[B5] Abdel-Razzak Z. Loyer P. Fautrel A. Gautier J. C. Corcos L. Turlin B. (1993). Cytokines down-regulate expression of major cytochrome P-450 enzymes in adult human hepatocytes in primary culture. *Mol. Pharmacol.* 44 707–715. 8232220

[B6] Andonegui G. Bonder C. S. Green F. Mullaly S. C. Zbytnuik L. Raharjo E. (2003). Endothelium-derived Toll-like receptor-4 is the key molecule in LPS-induced neutrophil sequestration into lungs. *J. Clin. Invest.* 111 1011–1020. 10.1172/JCI16510 12671050 PMC152584

[B7] Bjornsson E. S. Bergmann O. M. Bjornsson H. K. Kvaran R. B. Olafsson S. (2013). Incidence, presentation, and outcomes in patients with drug-induced liver injury in the general population of Iceland. *Gastroenterology* 144 1419–1425 1425.e1–1425.e3; quiz e19–e20. 10.1053/j.gastro.2013.02.006 23419359

[B8] Brady J. F. Li D. C. Ishizaki H. Yang C. S. (1988). Effect of diallyl sulfide on rat liver microsomal nitrosamine metabolism and other monooxygenase activities. *Cancer Res.* 48 5937–5940. 3167846

[B9] Brady J. F. Wang M. H. Hong J. Y. Xiao F. Li Y. Yoo J. S. (1991). Modulation of rat hepatic microsomal monooxygenase enzymes and cytotoxicity by diallyl sulfide. *Toxicol. Appl. Pharmacol.* 108 342–354. 10.1016/0041-008X(91)90123-V 2017758

[B10] Caulfield A. J. Wengenack N. L. (2016). Diagnosis of active tuberculosis disease: from microscopy to molecular techniques. *J. Clin. Tuberc. Other Mycobact. Dis.* 4 33–43. 10.1016/j.jctube.2016.05.005 PMC6850262 31723686

[B11] Cederbaum A. I. Yang L. Wang X. Wu D. (2012). CYP2E1 sensitizes the liver to LPS- and TNF alpha-induced toxicity via elevated oxidative and nitrosative stress and activation of ASK-1 and JNK mitogen-activated kinases. *Int. J. Hepatol.* 2012:582790. 10.1155/2012/582790 22028977 PMC3199085

[B12] Cervantes J. (2016). Tuberculosis. Digging deep in the soul of humanity. *Respir. Med.* 119 20–22. 10.1016/j.rmed.2016.08.009 27692142

[B13] Cheng J. Krausz K. W. Li F. Ma X. Gonzalez F. J. (2013). CYP2E1-dependent elevation of serum cholesterol, triglycerides, and hepatic bile acids by isoniazid. *Toxicol. Appl. Pharmacol.* 266 245–253. 10.1016/j.taap.2012.10.024 23142471 PMC3661416

[B14] Cui N. Wang H. Long Y. Su L. Liu D. (2015). Dexamethasone suppressed LPS-induced matrix metalloproteinase and its effect on endothelial glycocalyx shedding. *Mediators Inflamm.* 2015:912726. 10.1155/2015/912726 26199464 PMC4493300

[B15] Dandona P. Mohanty P. Hamouda W. Aljada A. Kumbkarni Y. Garg R. (1999). Effect of dexamethasone on reactive oxygen species generation by leukocytes and plasma interleukin-10 concentrations: a pharmacodynamic study. *Clin. Pharmacol. Ther.* 66 58–65. 10.1016/S0009-9236(99)70054-8 10430110

[B16] Davenport D. M. Wargovich M. J. (2005). Modulation of cytochrome P450 enzymes by organosulfur compounds from garlic. *Food Chem. Toxicol.* 43 1753–1762. 10.1016/j.fct.2005.05.018 16000231

[B17] Deng X. Luyendyk J. P. Ganey P. E. Roth R. A. (2009). Inflammatory stress and idiosyncratic hepatotoxicity: hints from animal models. *Pharmacol. Rev.* 61 262–282. 10.1124/pr.109.001727 19805476 PMC2763781

[B18] Eum S. Y. Maghni K. Hamid Q. Eidelman D. H. Campbell H. Isogai S. (2003). Inhibition of allergic airways inflammation and airway hyperresponsiveness in mice by dexamethasone: role of eosinophils, IL-5, eotaxin, and IL-13. *J. Allergy Clin. Immunol.* 111 1049–1061. 10.1067/mai.2003.1416 12743570

[B19] Fan M. Wang X. Xu G. Yan Q. Huang W. (2014). Bile acid signaling and liver regeneration. *Biochim. Biophys. Acta* 1849 196–200. 10.1016/j.bbagrm.2014.05.021 24878541 PMC4246016

[B20] Grudzinski I. P. Frankiewicz-Jozko A. Bany J. (2001). Diallyl sulfide–a flavour component from garlic (*Allium sativum*) attenuates lipid peroxidation in mice infected with *Trichinella spiralis*. *Phytomedicine* 8 174–177. 10.1078/0944-7113-00037 11417909

[B21] Hakkola J. Hu Y. Ingelman-Sundberg M. (2003). Mechanisms of down-regulation of CYP2E1 expression by inflammatory cytokines in rat hepatoma cells. *J. Pharmacol. Exp. Ther.* 304 1048–1054. 10.1124/jpet.102.041582 12604681

[B22] Hansson T. Tindberg N. Ingelman-Sundberg M. Kohler C. (1990). Regional distribution of ethanol-inducible cytochrome P450 IIE1 in the rat central nervous system. *Neuroscience* 34 451–463. 10.1016/0306-4522(90)90154-V 2333153

[B23] Hassan H. A. Hafez H. S. Zeghebar F. E. (2010). Garlic oil as a modulating agent for oxidative stress and neurotoxicity induced by sodium nitrite in male albino rats. *Food Chem. Toxicol.* 48 1980–1985. 10.1016/j.fct.2010.05.001 20457208

[B24] Hassan H. M. Guo H. Yousef B. A. Guerram M. Hamdi A. M. Zhang L. (2016). Role of inflammatory and oxidative stress, cytochrome P450 2E1, and bile acid disturbance in rat liver injury induced by isoniazid and lipopolysaccharide cotreatment. *Antimicrob. Agents Chemother.* 60 5285–5293. 10.1128/AAC.00854-16 27324775 PMC4997853

[B25] Hassan H. M. Guo H. Yousef B. A. Ping-Ping D. Zhang L. Jiang Z. (2017). Dexamethasone pretreatment alleviates isoniazid/lipopolysaccharide hepatotoxicity: inhibition of inflammatory and oxidative stress. *Front. Pharmacol.* 8:133. 10.3389/fphar.2017.00133 28360859 PMC5350150

[B26] Hassan H. M. Guo H. L. Yousef B. A. Luyong Z. Zhenzhou J. (2015). Hepatotoxicity mechanisms of isoniazid: a mini-review. *J. Appl. Toxicol.* 35 1427–1432. 10.1002/jat.3175 26095833

[B27] Heo B. G. Park Y. S. Chon S. U. Lee S. Y. Cho J. Y. Gorinstein S. (2007). Antioxidant activity and cytotoxicity of methanol extracts from aerial parts of Korean salad plants. *Biofactors* 30 79–89. 10.1002/biof.5520300202 18356580

[B28] Ho C. Y. Cheng Y. T. Chau C. F. Yen G. C. (2012). Effect of diallyl sulfide on *in vitro* and *in vivo* Nrf2-mediated pulmonic antioxidant enzyme expression via activation ERK/p38 signaling pathway. *J. Agric. Food Chem.* 60 100–107. 10.1021/jf203800d 22118872

[B29] Ho C. Y. Weng C. J. Jhang J. J. Cheng Y. T. Huang S. M. Yen G. C. (2014). Diallyl sulfide as a potential dietary agent to reduce TNF-alpha- and histamine-induced proinflammatory responses in A7r5 cells. *Mol. Nutr. Food Res.* 58 1069–1078. 10.1002/mnfr.201300617 24415531

[B30] Iranloye B. O. Oludare G. O. (2011). Garlic and vitamin E provides antioxidant defence in tissues of female rats treated with nicotine. *Niger. J. Physiol. Sci.* 26 103–107. 22314996

[B31] Jahan S. Khan M. Imran S. Sair M. (2015). The hepatoprotective role of Silymarin in isoniazid induced liver damage of rabbits. *J. Pak. Med. Assoc.* 65 620–622. 26060158

[B32] Jin L. Baillie T. A. (1997). Metabolism of the chemoprotective agent diallyl sulfide to glutathione conjugates in rats. *Chem. Res. Toxicol.* 10 318–327. 10.1021/tx9601768 9084912

[B33] Kalayarasan S. Prabhu P. N. Sriram N. Manikandan R. Arumugam M. Sudhandiran G. (2009). Diallyl sulfide enhances antioxidants and inhibits inflammation through the activation of Nrf2 against gentamicin-induced nephrotoxicity in Wistar rats. *Eur. J. Pharmacol.* 606 162–171. 10.1016/j.ejphar.2008.12.055 19374873

[B34] Kalayarasan S. Sriram N. Sudhandiran G. (2008). Diallyl sulfide attenuates bleomycin-induced pulmonary fibrosis: critical role of iNOS, NF-kappaB, TNF-alpha and IL-1beta. *Life Sci.* 82 1142–1153. 10.1016/j.lfs.2008.03.018 18462759

[B35] Karthikeyan S. (2004). Hepatotoxicity of isoniazid: a study on the activity of marker enzymes of liver toxicity in serum and liver tissue of rabbits. *Indian J. Pharmacol.* 36 244–250.

[B36] Kelicen P. Tindberg N. (2004). Lipopolysaccharide induces CYP2E1 in astrocytes through MAP kinase kinase-3 and C/EBPbeta and -delta. *J. Biol. Chem.* 279 15734–15742. 10.1074/jbc.M311850200 14670949

[B37] Khalili H. Dashti-Khavidaki S. Rasoolinejad M. Rezaie L. Etminani M. (2009). Anti-tuberculosis drugs related hepatotoxicity; incidence, risk factors, pattern of changes in liver enzymes and outcome. *DARU* 17 163–167.

[B38] Kozlov A. V. Duvigneau J. C. Miller I. Nurnberger S. Gesslbauer B. Kungl A. (2009). Endotoxin causes functional endoplasmic reticulum failure, possibly mediated by mitochondria. *Biochim. Biophys. Acta* 1792 521–530. 10.1016/j.bbadis.2009.03.004 19327397

[B39] Lee D. Y. Li H. Lim H. J. Lee H. J. Jeon R. Ryu J.-H. (2012). Anti-inflammatory activity of sulfur-containing compounds from garlic. *J. Med. Food* 15 992–999. 10.1089/jmf.2012.2275 23057778 PMC3491620

[B40] Lee H. S. Lee C. H. Tsai H. C. Salter D. M. (2009). Inhibition of cyclooxygenase 2 expression by diallyl sulfide on joint inflammation induced by urate crystal and IL-1beta. *Osteoarthritis Cartilage* 17 91–99. 10.1016/j.joca.2008.05.010 18573668

[B41] Liu J. Sendelbach L. E. Parkinson A. Klaassen C. D. (2000). Endotoxin pretreatment protects against the hepatotoxicity of acetaminophen and carbon tetrachloride: role of cytochrome P450 suppression. *Toxicology* 147 167–176. 10.1016/S0300-483X(00)00193-1 10924799

[B42] Livak K. J. Schmittgen T. D. (2001). Analysis of relative gene expression data using real-time quantitative PCR and the 2^-ΔΔC_T_^ method. *Methods* 25 402–408. 10.1006/meth.2001.1262 11846609

[B43] Loida P. J. Sligar S. G. (1993). Molecular recognition in cytochrome P-450: mechanism for the control of uncoupling reactions. *Biochemistry* 32 11530–11538. 10.1021/bi00094a009 8218220

[B44] Lu J. Jones A. D. Harkema J. R. Roth R. A. Ganey P. E. (2011). Amiodarone exposure during modest inflammation induces idiosyncrasy-like liver injury in rats: role of tumor necrosis factor-alpha. *Toxicol. Sci.* 125 126–133. 10.1093/toxsci/kfr266 21984482 PMC3243747

[B45] Lu Y. Cederbaum A. I. (2008). CYP2E1 and oxidative liver injury by alcohol. *Free Radic. Biol. Med.* 44 723–738. 10.1016/j.freeradbiomed.2007.11.004 18078827 PMC2268632

[B46] Lu Y. Cederbaum A. I. (2010). CYP2E1 potentiation of LPS and TNFalpha-induced hepatotoxicity by mechanisms involving enhanced oxidative and nitrosative stress, activation of MAP kinases, and mitochondrial dysfunction. *Genes Nutr.* 5 149–167. 10.1007/s12263-009-0150-5 19798529 PMC2885169

[B47] Luyendyk J. P. Shores K. C. Ganey P. E. Roth R. A. (2002). Bacterial lipopolysaccharide exposure alters aflatoxin B(1) hepatotoxicity: benchmark dose analysis for markers of liver injury. *Toxicol. Sci.* 68 220–225. 10.1093/toxsci/68.1.220 12075124

[B48] Metushi I. G. Nakagawa T. Uetrecht J. (2012). Direct oxidation and covalent binding of isoniazid to rodent liver and human hepatic microsomes: humans are more like mice than rats. *Chem. Res. Toxicol.* 25 2567–2576. 10.1021/tx300341r 23016703 PMC3501148

[B49] Mörk L.-M. Strom S. C. Mode A. Ellis E. C. S. (2016). Addition of dexamethasone alters the bile acid composition by inducing CYP8B1 in primary cultures of human hepatocytes. *J. Clin. Exp. Hepatol.* 6 87–93. 10.1016/j.jceh.2016.01.007 27493455 PMC4963319

[B50] Neyrinck A. M. Mouson A. Delzenne N. M. (2007). Dietary supplementation with laminarin, a fermentable marine beta (1-3) glucan, protects against hepatotoxicity induced by LPS in rat by modulating immune response in the hepatic tissue. *Int. Immunopharmacol.* 7 1497–1506. 10.1016/j.intimp.2007.06.011 17920526

[B51] Pedraza-Chaverri J. Gonzalez-Orozco A. E. Maldonado P. D. Barrera D. Medina-Campos O. N. Hernandez-Pando R. (2003a). Diallyl disulfide ameliorates gentamicin-induced oxidative stress and nephropathy in rats. *Eur. J. Pharmacol.* 473 71–78. 12877940 10.1016/s0014-2999(03)01948-4

[B52] Pedraza-Chaverri J. Maldonado P. D. Barrera D. Ceron A. Medina-Campos O. N. Hernandez-Pando R. (2003b). Protective effect of diallyl sulfide on oxidative stress and nephrotoxicity induced by gentamicin in rats. *Mol. Cell. Biochem.* 254 125–130. 14674690 10.1023/a:1027372102135

[B53] Ramaiah S. K. Apte U. Mehendale H. M. (2001). Cytochrome P4502E1 induction increases thioacetamide liver injury in diet-restricted rats. *Drug Metab. Dispos.* 29 1088–1095. 11454726

[B54] Ramappa V. Aithal G. P. (2013). Hepatotoxicity related to anti-tuberculosis drugs: mechanisms and management. *J. Clin. Exp. Hepatol.* 3 37–49. 10.1016/j.jceh.2012.12.001 25755470 PMC3940184

[B55] Rao P. S. Midde N. M. Miller D. D. Chauhan S. Kumar A. Kumar S. (2015). Diallyl sulfide: potential use in novel therapeutic interventions in alcohol, drugs, and disease mediated cellular toxicity by targeting cytochrome P450 2E1. *Curr. Drug Metab.* 16 486–503. 26264202 10.2174/1389200216666150812123554 PMC4663692

[B56] Rendic S. P. (1999). Cytochrome P450 enzymes (CYP ENZYMES): role in toxic effects of xenobiotics. *Biochem. Med.* 9 107–113.

[B57] Roth R. A. Harkema J. R. Pestka J. P. Ganey P. E. (1997). Is exposure to bacterial endotoxin a determinant of susceptibility to intoxication from xenobiotic agents? *Toxicol. Appl. Pharmacol.* 147 300–311. 10.1006/taap.1997.8301 9439725

[B58] Shayakhmetova G. M. Bondarenko L. B. Voronina A. K. Anisimova S. I. Matvienko A. V. Kovalenko V. M. (2015). Induction of CYP2E1 in testes of isoniazid-treated rats as possible cause of testicular disorders. *Toxicol. Lett.* 234 59–66. 10.1016/j.toxlet.2015.02.008 25683034

[B59] Shu C. C. Lee C. H. Lee M. C. Wang J. Y. Yu C. J. Lee L. N. (2013). Hepatotoxicity due to first-line anti-tuberculosis drugs: a five-year experience in a Taiwan medical centre. *Int. J. Tuberc. Lung Dis.* 17 934–939. 10.5588/ijtld.12.0782 23743313

[B60] Singh M. Sasi P. Rai G. Gupta V. Amarapurkar D. Wangikar P. (2011). Studies on toxicity of antitubercular drugs namely isoniazid, rifampicin, and pyrazinamide in an in vitro model of HepG2 cell line. *Med. Chem. Res.* 20 1611–1615. 10.1007/s00044-010-9405-3

[B61] Steele M. A. Burk R. F. Desprez R. M. (1991). Toxic hepatitis with isoniazid and rifampin. A meta-analysis. *Chest* 99 465–471. 10.1378/chest.99.2.465 1824929

[B62] Su Y. Zhang Y. Chen M. Jiang Z. Sun L. Wang T. (2014). Lipopolysaccharide exposure augments isoniazide-induced liver injury. *J. Appl. Toxicol.* 34 1436–1442. 10.1002/jat.2979 25331106

[B63] Sung J. Harfouche Y. De La Cruz M. Zamora M. P. Liu Y. Rego J. A. (2015). Garlic (*Allium sativum*) stimulates lipopolysaccharide-induced tumor necrosis factor-alpha production from J774A.1 murine macrophages. *Phytother. Res.* 29 288–294. 10.1002/ptr.5253 25366263

[B64] Szabo G. Csak T. (2012). Inflammasomes in liver diseases. *J. Hepatol.* 57 642–654. 10.1016/j.jhep.2012.03.035 22634126

[B65] Tomlinson J. E. Blikslager A. T. (2004). Interactions between lipopolysaccharide and the intestinal epithelium. *J. Am. Vet. Med. Assoc.* 224 1446–1452. 10.2460/javma.2004.224.1446 15124884

[B66] Tostmann A. Boeree M. J. Peters W. H. Roelofs H. M. Aarnoutse R. E. Van Der Ven A. J. (2008). Isoniazid and its toxic metabolite hydrazine induce *in vitro* pyrazinamide toxicity. *Int. J. Antimicrob. Agents* 31 577–580. 10.1016/j.ijantimicag.2008.01.022 18358703

[B67] Wang K. (2014). Molecular mechanisms of liver injury: apoptosis or necrosis. *Exp. Toxicol. Pathol.* 66 351–356. 10.1016/j.etp.2014.04.004 24867271

[B68] White R. E. (1991). The involvement of free radicals in the mechanisms of monooxygenases. *Pharmacol. Ther.* 49 21–42. 10.1016/0163-7258(91)90020-M 1852787

[B69] Whitney J. B. Wainberg M. A. (2002). Isoniazid, the frontline of resistance in *Mycobacterium tuberculosis*. *McGill J. Med.* 6 114–123.

[B70] Xu J. Ma H.-Y. Liang S. Sun M. Karin G. Koyama Y. (2017). The role of human cytochrome P450 2E1 in liver inflammation and fibrosis. *Hepatol. Commun.* 1 1043–1057. 10.1002/hep4.1115 29404441 PMC5721400

[B71] Yang C. S. Chhabra S. K. Hong J. Y. Smith T. J. (2001). Mechanisms of inhibition of chemical toxicity and carcinogenesis by diallyl sulfide (DAS) and related compounds from garlic. *J. Nutr.* 131 1041S–1045S. 10.1093/jn/131.3.1041S 11238812

[B72] Yin M. C. Hwang S. W. Chan K. C. (2002). Nonenzymatic antioxidant activity of four organosulfur compounds derived from garlic. *J. Agric. Food Chem.* 50 6143–6147. 10.1021/jf0204203 12358493

[B73] Yue J. Peng R. X. Yang J. Kong R. Liu J. (2004). CYP2E1 mediated isoniazid-induced hepatotoxicity in rats. *Acta Pharmacol. Sin.* 25 699–704. 15132840

[B74] Zumla A. I. Gillespie S. H. Hoelscher M. Philips P. P. J. Cole S. T. Abubakar I. (2014). New antituberculosis drugs, regimens, and adjunct therapies: needs, advances, and future prospects. *Lancet Infect. Dis.* 14 327–340. 10.1016/S1473-3099(13)70328-1 24670627

