# Supplementary material for: Investigating the CYP2E1 Potential Role in the Mechanisms Behind INH/LPS-Induced Hepatotoxicity
Source: Front Pharmacol. 2018 Mar 7;9:198. doi: 10.3389/fphar.2018.00198 (PMC5850051; doi:10.3389/fphar.2018.00198)
Supplement: Supplementary file 1 [file Table_1.DOCX]

**Table S1 Primers used for Real-time PCR**

| Gene | Primer orientation | Nucleotide sequence | NCBI Gene ID number |
| --- | --- | --- | --- |
| *GAPDH* | forward | 5’- ATGGAGAAGGCTGGGGCTCACCT-3’ | 24383 |
|  | reverse | 5’-AGCCCTTCCACGATGCCAAAGTTGT-3’ |  |
| *SOD1* | forward | 5’-AGGGCGTCATTCACTTCGAG-3’ | 24786 |
|  | reverse | 5’-CCTCTCTTCATCCGCTGGAC-3’ |  |
| *SOD2* | forward | 5’-GCCTCAGCAATGTTGTGTCG-3’ | 24787 |
|  | reverse | 5’-ATTGTTCACGTAGGTCGCGT-3’ |  |
| *GPX1* | forward | 5’-CGGACATCAGGAGAATGGCA-3’ | 24404 |
|  | reverse | 5’-GTAAAGAGCGGGTGAGCCTT-3’ |  |
| *GPX4* | forward | 5’-GCCGTCTGAGCCGCTTATT-3’ | 29328 |
|  | reverse | 5’-CGATGTCCTTGGCTGCGAAT-3’ |  |
| *FXR* | forward | 5’- TGGACTCATACAGCAAACAGAGA-3’ | 60351 |
|  | reverse | 5’- GTCTGAAACCCTGGAAGTCTTTT-3’ |  |
| *SHP* | forward | 5’- ACCTGCAACAGGAGGCTCACT-3’ | 117274 |
|  | reverse | 5’- TGGAAGCCATGAGGAGGATTC-3’ |  |
| *CYP7A1* | forward | 5’- CAGGGAGATGCTCTGTGTTCA-3’ | 25428 |
|  | reverse | 5’- AGGCATACATCCCTTCCGTGA-3’ |  |
| *CYP27A1* | forward | 5’- GGAAGGTGCCCCAGAACAA-3’ | 301517 |
|  | reverse | 5’- GCGCAGGGTCTCCTTAATCA-3’ |  |
| *CYP8B1* | forward | 5’- GTACACATGGACCCCGACATC-3’ | 81924 |
|  | reverse | 5’- GGGTGCCATCAGGGTTGAG-3’ |  |
| *BSEP* | forward | 5’- CAACGCATTGCTATTGCTCG-3’ | 83569 |
|  | reverse | 5’- CTTCTGGATGGTGGACAAACG-3’ |  |
| *NTCP* | forward | 5’- GCATGATGCCACTCCTCTTATAC-3’ | 24777 |
|  | reverse | 5’- TACATAGTGTGGCCTTTTGGACT-3’ |  |
| *MRP2* | forward | 5’- CAGTCACGGCTTCCTTTCTG-3’ | 25303 |
|  | reverse | 5’- AGGTTTCCGCTGGGACTTCT-3’ |  |
| *MRP3* | forward | 5’- ACACCGAGCCAGCCATATAC-3’ | 140668 |
|  | reverse | 5’- TCAGCTTCACATTGCCTGTC-3’ |  |
| *OATP1* | forward | 5’- GGCTTTTTGGTCTGTGCAGG-3’ | 50572 |
|  | reverse | 5’- CACCTTGTGTTGCAGTCAGC-3’ |  |
| *OATP2* | forward | 5’-TGTGATGACCGTTGATAATTTTCCA-3’ | 170698 |
|  | reverse | 5’-TTCTCCACATATAGTTGGTGCTGAA-3’ |  |
| *TNFα* | forward | 5’- TGGTGGGAGACATTGGAGAT-3’ | 24835 |
|  | reverse | 5’- GCGGCTTGATAAACACATCA-3’ |  |
| *IL-6* | forward | 5’- AGAGACTTCCAGCCAGTTGC-3’ | 24498 |
|  | reverse | 5’- AGTCTCCTCTCCGGACTTGT-3’ |  |
| *IL-1α* | forward | 5’- CCTCGTCCTAAGTCACTCGC-3’ | 24493 |
|  | reverse | 5’- GGCTGGTTCCACTAGGCTTT-3’ |  |
| *IL-1β* | forward | 5’- CCTGTTCTTTGAGGCTGACA-3’ | 24494 |
|  | reverse | 5’- GCTGTGAGATTTGAAGCTGGA-3’ |  |
| *INFγ* | forward | 5’- ACAACCCACAGATCCAGCAC-3’ | 25712 |
|  | reverse | 5’- CCAGAATCAGCACCGACTCC-3’ |  |
| *CYP2E1* | forward | 5’- GTGGTCCTGCATGGCTACA-3’ | 25086 |
|  | reverse | 5’- ACCTCCGCACATCCTTCC-3’ |  |
| *PPARα* | forward | 5’- GTCCTCTGGTTGTCCCCTTG-3’ | 25747 |
|  | reverse | 5’- GTCAGTTCACAGGGAAGGCA-3’ |  |
| *FAS* | forward | 5’- GACTACAGACAACAGCAACC-3’ | 50671 |
|  | reverse | 5’- CTCAGACAGGCACTCAGG-3’ |  |
| *HMGCS* | forward | 5’- GGACCAACCTTCTACCTCAG-3’ | 29637 |
|  | reverse | 5’- ACAACTCACCAGCCATCAC-3’ |  |
