# Supplementary material for: Investigating the CYP2E1 Potential Role in the Mechanisms Behind INH/LPS-Induced Hepatotoxicity
Source: Front Pharmacol. 2018 Mar 7;9:198. doi: 10.3389/fphar.2018.00198 (PMC5850051; doi:10.3389/fphar.2018.00198)
Supplement: Supplementary file 2 [file Table_2.DOCX]

**Table S2 Antibodies for Western blot**

| **Primary antibodies** | | |
| --- | --- | --- |
| Antibody | Catalog number | Company |
| *FXR* | bs-12867R | Bioss, Atlanta, GA, USA |
| *CYP8B1* | sc-23515 | Santa Cruz, CA, USA |
| *BSEP* | sc-25571 | Santa Cruz, CA, USA |
| *NTCP* | sc-98485 | Santa Cruz, CA, USA |
| *CYP2E1* | ab19140 | Abcam, Cambridge, UK |
| *PPARα* | sc-9000 | Santa Cruz, CA, USA |
| *Cleaved caspase 3* | 9661 | Cell Signaling Technology (CST) Danvers, MA, USA |
| *β-actin* | sc-69879 | Santa Cruz, CA, USA |
| \| **Secondary antibodies** \| \| \| \| --- \| --- \| --- \| \| Antibody \| Catalog number \| Company \| \| *Goat anti-mouse* \| ab6789 \| Abcam, Cambridge, UK \| \| *Goat anti-rabbit* \| ab6721 \| Abcam, Cambridge, UK \| \| *Rabbit anti-goat* \| ab6741 \| Abcam, Cambridge, UK \| | | |
